# Overestimation of the effect of (fos)aprepitant on intravenous dexamethasone pharmacokinetics requires adaptation of the guidelines for children with chemotherapy-induced nausea and vomiting

**DOI:** 10.1007/s00520-022-07423-6

**Published:** 2022-10-26

**Authors:** A. Laura Nijstad, Evelien de Vos-Kerkhof, Catherine F. Enters-Weijnen, Marianne D. van de Wetering, Wim J. E. Tissing, Matthijs M. Tibben, Hilde Rosing, Arief Lalmohamed, Alwin D. R. Huitema, C. Michel Zwaan

**Affiliations:** 1 Department of Clinical Pharmacy, University Medical Center Utrecht, Utrecht, The Netherlands; 2 Department of Pharmacology, Princess Máxima Center for Pediatric Oncology, Utrecht, The Netherlands; 3 Princess Máxima Center for Pediatric Oncology, Postbus 113, 3720 AC Bilthoven, Utrecht, The Netherlands; 4 Julius Center for Health Sciences and Primary Care, University Medical Center Utrecht, Utrecht, The Netherlands; 5 Department of Pediatric Oncology and Hematology, University of Groningen, University Medical Center Groningen, Groningen, The Netherlands; 6 Department of Pharmacy & Pharmacology, Netherlands Cancer Institute, Amsterdam, The Netherlands; 7 Utrecht Institute for Pharmaceutical Sciences, Utrecht University, Utrecht, The Netherlands; 8 Department of Pediatric Oncology, Erasmus MC-Sophia Children’s Hospital, Rotterdam, The Netherlands

**Keywords:** Antiemetics, Childhood cancer, Oncology, Pediatrics, Pharmacokinetics

## Abstract

**Purpose:**

Chemotherapy-induced nausea and vomiting (CINV) are common side effects in pediatric oncology treatment. Besides 5-HT_3_-antagonists, both dexamethasone and aprepitant are cornerstone drugs in controlling these side effects. Based on results of adult studies, the dexamethasone dose is reduced by 50% when combined with aprepitant, because of a drug-drug interaction, even though data on the interaction in children is lacking. The current study was developed to investigate the effect of aprepitant on dexamethasone clearance (CL) in children, in order to assess if dexamethasone dose reduction for concomitant use of aprepitant is appropriate in the current antiemetic regimen.

**Methods:**

In total, 65 children (0.6–17.9 years), receiving intravenous or oral antiemetic therapy (dexamethasone ± aprepitant) as standard of care, were included. 305 dexamethasone plasma concentrations were determined using LC–MS/MS. An integrated dexamethasone and aprepitant pharmacokinetic model was developed using non-linear mixed effects modelling in order to investigate the effect of aprepitant administration on dexamethasone CL.

**Results:**

In this population, dexamethasone CL in patients with concomitant administration of aprepitant was reduced by approximately 30% of the uninhibited CL (23.3 L/h (95% confidence interval 20.4–26.0)). This result is not consistent with the results of adult studies (50% reduction). This difference was not age dependent, but might be related to the route of administration of dexamethasone. Future studies are needed to assess the difference in oral/intravenous dexamethasone.

**Conclusion:**

When dexamethasone is given intravenously as a component of triple therapy to prevent CINV in children, we advise to reduce the dexamethasone dose by 30% instead of 50%.

**Supplementary Information:**

The online version contains supplementary material available at 10.1007/s00520-022-07423-6.

## Introduction

Chemotherapy-induced nausea and vomiting (CINV) are one of the most common side effects of pediatric oncology treatment. International guidelines for management and prevention of these side effects have been developed [1–4]. Different treatment levels are based on the emetogenicity classification of the Canadian Pediatric Oncology Group of Ontario [1, 5]. The overall backbone of antiemetic supportive care consist of a 5-HT_3_ receptor antagonist for low emetogenic chemotherapy, addition of dexamethasone for moderate emetogenic chemotherapy (MEC) and, for highly emetogenic chemotherapy (HEC), addition of the NK1 receptor antagonist, aprepitant.

Dexamethasone shows a bioavailability of approximately 86% after oral administration [6]. It is rapidly distributed and about 77% is bound to plasma proteins [7]. Dexamethasone is metabolized by cytochrome P450-3A4 (CYP3A4)[7], the elimination half-life is 3–6 h for adults which is higher in children or infants (3–8 h and 2–10 h, respectively) [8]. Aprepitant is administered orally and shows a bioavailability of 59–67%. Aprepitant is highly bound to plasma proteins (97%). The metabolism of aprepitant occurs mainly via oxidation and CYP3A4, and the half-life is 9–13 h in adults [9, 10]. Fosaprepitant is a water soluble prodrug, the intravenous formulation of aprepitant. Within 30 min after administration, fosaprepitant is converted to aprepitant, after which it shows similar pharmacokinetics (PK) [10].

Besides being a substrate for CYP3A4, (fos)aprepitant is a weak to moderate inhibitor of CYP3A4. (Fos)aprepitant therapy can result in higher plasma levels of CYP3A4 metabolized agents (e.g. dexamethasone). In adult studies, it has been shown that concurrent use of aprepitant resulted in lower dexamethasone clearances (CL) and a higher exposure to dexamethasone of approximately twofold, and thus, a 50% dexamethasone dose reduction is recommended [11–14]. However, for children, this reduction is based on extrapolation of adult data, and it is not known if the magnitude of this interaction is the same [15].

Moreover, despite standardized prophylaxis, children receiving HEC achieve 20–30% less CINV control compared to adults[16–18], which might be explained by suboptimal dexamethasone therapy, due to a possible different interaction in children compared to adults. (Fos)aprepitant and dexamethasone doses used in children are different than in adults, which could lead to a different magnitude of the interaction. Moreover, developmental changes occur over age (e.g. development of liver enzymes), which could lead to altered PK in young children [19].

In this study, we studied the effect of aprepitant on dexamethasone PK in pediatric oncology patients, in order to assess whether dexamethasone dose reduction for concomitant aprepitant use is appropriate in the current antiemetic regimen.

## Methods

### Patients, sampling, and bioanalysis

A prospective observational study was performed in Princess Máxima Center for Pediatric Oncology, the Netherlands. Patients aged 0.5–18 years with a new oncological diagnosis, having a central venous line, planned to receive chemotherapy with granisetron/ondansetron; dexamethasone and/or aprepitant as standard of care were screened for eligibility. Patients could be included twice: once for oral aprepitant and once for intravenous fosaprepitant, if applicable. Patients using strong CYP3A4-substrates and/or -inhibitors within 7 days or strong CYP3A4-inducers within 30 days before the start of antiemetic therapy were excluded (Supplementary Table S1). Patients with Down syndrome were excluded due to possible altered PK as compared to children without Down syndrome. Written informed consent was obtained prior to participation. Ethical approval by the institutional Medical Ethics Committee of the Erasmus MC was obtained. The study was registered in the Dutch Trial Registry as NTR7720.

Patients were treated with dexamethasone with (HEC) or without (MEC) (fos)aprepitant according to the antiemetic guidelines of the Dutch Childhood Oncology Group (Table 1). Blood samples of 1 mL were collected from the central venous line before and after administration of the antiemetics on day 1, 2, or 3. A pre-dose sample was taken when dexamethasone was administered in the previous 7 days. Post-dose, a maximum of six samples were taken over 24 h (Supplementary Table S2). The exact number of collected samples depended on the availability of the central venous line (especially in patients with a single lumen line and continuous chemotherapy infusions). Patients with at least one sample post-dose were included in the analysis.

**Table 1:** Dose regimens for dexamethasone and (fos)aprepitant

Emetogenicity	Dexamethasone^1^	Aprepitant^2^	Fosaprepitant^3^
Moderate	< 0.6 m^2^: 2 mg BID iv/po > 0.6 m^2^: 4 mg BID iv/po		
High	3 mg/m^2^ QID iv/po (or 6 mg/m^2^ QID iv/po without aprepitant)	Day 1: 3 mg/kg QD po (max 125 mg) Day 2–3: 2 mg/kg QD po (max 80 mg)	Day 1: 3 mg/kg QD iv (max 115 mg) Day 2–3: 2 mg/kg QD iv (max 80 mg)

*BID*, twice daily; *QD*, once daily; *QID*, four times daily

^1^Dexamethasone was administered as commercially available tablets, oral solution, or solution for injection, using locally available products

^2^Aprepitant was administered as 80 mg or 125 mg capsules (Merck Sharp & Dohme B.V. [9] or generic equivalent) or as an extemporaneous oral suspension of 10 mg/mL (prepared using the commercially available aprepitant capsule filling and a suspension base as described by Nijstad et al. [28])

^3^Fosaprepitant was administered using the commercially available iv fosaprepitant formulation (Ivemend, Merck Sharp & Dohme B.V. [10])

Plasma samples were stored at − 80 °C until analyzation. Plasma concentrations of dexamethasone and aprepitant were measured using a validated liquid chromatography mass spectrometry method, with a lower limit of quantification (LLOQ) of 1 µg/L and 0.1 µg/L respectively, as described previously [20]. The first post-dose samples below LLOQ were included as a plasma concentration of 0.5 µg/L (1/2 LLOQ for dexamethasone); other samples below LLOQ were omitted [21].

### Sample size

In order to study age-related differences, children ≤ 18 years were divided in three age groups (≥ 0.5–6 years; ≥ 6–12 years and ≥ 12 years) treated with dexamethasone with/without (fos)aprepitant. We planned to include 30 children in each age category, with the aim to distinguish 15 children using dexamethasone without (fos)aprepitant and 15 children using dexamethasone with (fos)aprepitant. We planned to evaluate at least 5 children across all age groups, based on previous experience in similar projects.

### Dexamethasone model development

Starting point for model development for dexamethasone were one- and two-compartment models with first-order absorption [22–25]. Allometric scaling using body weight was a priori included on all parameters [26].

Interindividual variability (IIV) was evaluated for all parameters, using an exponential function [27]. Since data of multiple dexamethasone doses were available (sampling over 24 h, while dexamethasone was administered twice or four times daily), interoccasion variability (IOV) was implemented similarly as IIV, with each dose and subsequent sampling defined as a separate occasion. This variability was evaluated for all parameters to diagnose potential time-dependent trends and allow for random unaccounted variability between dosing moments. Residual unexplained variability was evaluated as a proportional error model or as a combination of a proportional and additive error model.

### Dexamethasone covariate analysis

Following structural model development, the influence of patient-specific factors for variability in PK parameters was evaluated. Assessed covariates included age and (fos)aprepitant treatment to study the effect of (fos)aprepitant treatment on dexamethasone CL. Continuous covariates were evaluated using a power function. (Fos)aprepitant treatment was described as a binary categorical variable: 1 when (fos)aprepitant was administered concomitantly and 0 when no (fos)aprepitant was administered. (Fos)aprepitant as a covariate was tested on CL as follows:

$${\mathrm P}_{\mathrm i}={\mathrm P}_{\mathrm{pop}}\times{\mathrm P}_{\mathrm{cov}}^{(\mathrm{fos})\mathrm{aprepitant}}$$

where *P*_*cov*_ represents the estimated proportional factor by which CL changes at a specific covariate value.

### Integrated dexamethasone and aprepitant model development

In order to thoroughly investigate the drug-drug interaction, an integrated PK model of dexamethasone and (fos)aprepitant was developed. The PK of (fos)aprepitant was assumed to be described by a one-compartment model with absorption transit compartments, as described by Nijstad et al. [28]. The dataset that was used for the current model was previously used for the solely (fos)aprepitant model [28].

Two (hypothetical) enzyme compartments (enzyme_active_ and enzyme_inactive_) were added as previously described by Huitema et al. [29]. Elimination of dexamethasone was directly proportional to the amount of enzyme_active_ present in the compartment. The amount of enzyme_active_ was set to 1 and enzyme_inactive_ to 0 at *t* = 0. The conversion of enzyme_active_ to enzyme_inactive_ was driven by the amount of aprepitant in the central compartment.

### Model evaluation

Discrimination between models was guided by physiological plausibility, goodness-of-fit (GOF) plots, precision of parameter estimates, and change in objective function value (dOFV). A drop of ≥ 3.84 points, corresponding to a *P* < 0.05 (*χ*^2^ distribution with one degree of freedom (df)), was considered a significant improved fit for hierarchical models. The adequacy of the models was assessed by GOF plots and visual predictive checks (VPC) [30]. Parameter precision was assessed by the sampling importance resampling procedure [31].

### Drug-drug interaction between dexamethasone and aprepitant

Since patients in both groups (dexamethasone with/without (fos)aprepitant) were not treated with the same dexamethasone doses (Table 1), it is difficult to compare the observed areas under the curve (AUCs) between the two groups, in order to assess the influence of (fos)aprepitant on the AUC of dexamethasone. For this reason, simulations were carried out. Patients included in this study were used for the simulations and individuals were replicated to attain a total of 2000 patients per group. All patients were hypothetically treated, once with a single dose of intravenous dexamethasone alone and once with a single dose of intravenous dexamethasone plus oral aprepitant (doses according to Table 1, HEC day 1). In the first simulations, the hypothetical dexamethasone dose in both groups was the same (100%, 6 mg/m^2^), to compare AUCs. Subsequently, dexamethasone dose reductions (50% and 33%) were tested to test which dose regimen would lead to comparable (assessed visually and by range) AUCs for both groups. The AUC_t0-∞_ (in mg/L*h) was calculated using a dummy compartment in the final population PK model. The results of these simulated AUCs are used for the dexamethasone dose reduction recommendations.

### Software

Nonlinear mixed-effects modelling was performed using NONMEM (version 7.3.0, ICON development Solutions, Ellicott City, MD, USA) and Pearl-speaks-NONMEM (PsN, version 4.7.0) with First-Order Conditional Estimation with interaction (FOCE-I) as estimation method [32, 33]. Pirana (version 2.9.9) was used as graphical user interface for NONMEM [34]. R (version 3.4.3) was used for data handling and visualization [35].

## Results

### Patients and sampling

**Fig. 1:**
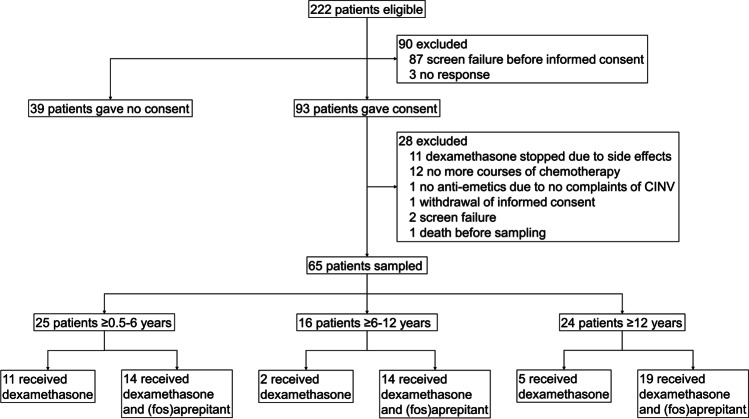
Trial profile

In total, 222 patients were eligible for inclusion in the study. Of these patients, 93 gave informed consent, 39 patients did not gave informed consent, 87 patients were screen failures before informed consent was given and 3 patients did not response before the study was closed. 65 of the 93 included patients with a median age of 8.8 years (range 0.6–17.9) were available for sampling in this study, and were included between March 2019 and April 2021. For trial profile, see Fig. 1. A total of 28 children were excluded after receiving informed consent. In 12 of these children, dexamethasone was stopped as antiemetic drug due to hypertension (*n* = 3), behavioral problems (*n* = 6), or a combination of those two (*n* = 1), bradycardia (*n* = 1) or no complaints of nausea (*n* = 1). No patients were included in the study twice.

Of the 65 sampled patients, 18 patients were treated with dexamethasone without aprepitant and 47 patients with dexamethasone plus aprepitant. We experienced a lower median age in the dexamethasone group compared to the dexamethasone with aprepitant group. In 80% of the patients, samples were taken on day 1 of antiemetic treatment. In total, 62 of the patients (95%) were treated with intravenous dexamethasone. Detailed patient characteristics are shown in Table 2. In total, 305 dexamethasone samples were available for analysis, of which 12 were below LLOQ (all single below LLOQ samples post-dose). No outliers were observed in our data. Supplementary Figure S1 displays the observed plasma concentrations over time.

**Table 2:** Patient characteristics, median (range)

	Dexamethasone without aprepitant	Dexamethasone with aprepitant	Total
	*N* = 18	*N* = 47	*N* = 65
Patient characteristics			
Female sex [*n* (%)]	8 (44%)	19 (40%)	27 (40%)
Age, years	3.9 (0.6–16.5)	10.0 (0.7–17.9)	8.8 (0.6–17.9)
Body weight, kg	16.3 (8.6–74.1)	32.5 (8.4–66.3)	29.5 (8.4–74.1)
Diagnosis			
Acute myeloid leukemia	0	3	3
Atypical teratoid rhabdoid tumor	1	1	2
Ependymoma	2	0	2
Ewing sarcoma	3	5	8
Glioma	2	0	2
Medulloblastoma	2	11	13
Neuroblastoma	1	7	8
Osteosarcoma	3	11	14
Rhabdomyosarcoma	0	5	5
Others	4	4	8
Dexamethasone iv [*n* (%)]	18 (100%)	44 (94%)	62 (95%)
Aprepitant po [*n* (%)]	NA	42 (89%)	42 (65%)
Available data			
Total no. of PK samples [*n*]	85	220	305
No of samples per patient	5 (1–6)	5 (1–7)	5 (1–7)

*NA*, not applicable

### Dexamethasone model development

A two-compartment model with first-order absorption was appropriate to describe the dexamethasone PK. The absorption rate constant (*k*_a_) could not be estimated due to the small number of patients that were treated with dexamethasone orally, so *k*_a_ was fixed to 1.5 h^−1^ [36, 37]. Interindividual variability (IIV) was included on CL, Q, and Vc, and interoccasion variability (IOV) on CL and Vc.

Inclusion of aprepitant as categorical covariate on CL led to an improved model fit (dOFV − 7.2). The proportional factor by which CL changes was estimated at 0.74 (95% confidence interval (CI) 0.56 − 1.01), signifying a 26% lower CL when aprepitant is administrated concomitantly.

### Integrated dexamethasone and aprepitant model development

The model was further optimized by integrating the dexamethasone and aprepitant models. A graphical representation of the combined model is showed in Supplementary Figure S3. The inhibition of dexamethasone CL was modelled by an aprepitant concentration-dependent reversible inhibition of enzymes. As mentioned in the methods, the total amount of enzymes consisted of an active and inactive part. Mass transport between enzyme_active_ and enzyme_inactive_ was modelled using an inhibition rate constant *k*_inh_ and a reactivation rate constant *k*_reac_. No age-related effects on any of the PK parameters were found.

The final model consisted of a two-compartment model with first-order absorption for dexamethasone, a one-compartment model with absorption transit compartments and two enzyme compartments describing the aprepitant concentration-dependent reversible inhibition of enzymes. The final estimates and 95%CI are shown in Table 3.

**Table 3:** Final dexamethasone population PK parameter estimates

PK parameter	Estimate	95% CI
${CL}_{dex,70kg} (L/h)$	23.3	20.4–26.0
${Vc}_{dex,70kg} (L)$	51.0	43.1–63.2
${Vp}_{dex,70kg} (L)$	42.5	36.2–47.4
${Q}_{dex,70kg} (L/h)$	47.6	40.1–57.9
*k*_inh_ (L/µg*h)	0.906	0.704–1.200
*k*_react_ (h^−1^)	1.30	0.888–1.90
IIV CL_dex_ (%)	36.6	29.5–43.8
IIV Vc_dex_ (%)	59.0	42.5–72.4
IIV Q_dex_ (%)	57.8	38.0–77.2
IOV CL_dex_ (%)	17.9	13.4–27.5
IOV Vc_dex_ (%)	14.4	5.6–23.3
Proportional residual error (%)	28.1	25.1–32.0

*PK*, pharmacokinetics; *CI*, confidence interval obtained by sampling importance resampling; *CL*, clearance; *Vc*, volume of distribution of the central compartment; *Vp*, volume of distribution of the peripheral compartment; *Q*, intercompartment clearance between Vc and Vp; *IIV*, interindividual variability; *IOV*, interoccassion variability

Population estimates CL_70kg_, Vc_70kg_, Vp_70kg_, and Q_70kg_ correspond to a subject weighing 70 kg and are adjusted to an individual value, using allometric scaling

The GOF plots (Supplementary Figure S3A and B) showed accurate population and individual predictions, without signs for over- or underprediction. CWRES are evenly distributed over the whole plasma concentration range (Supplementary Figure S3C) and time interval (Supplementary Figure S3D). The VPC demonstrated that the median and the 95%CI of the observed data were in line with those from the simulation-based predictions from the model (Supplementary Figure S4).

### Drug-drug interaction between dexamethasone and aprepitant

**Fig. 2:**
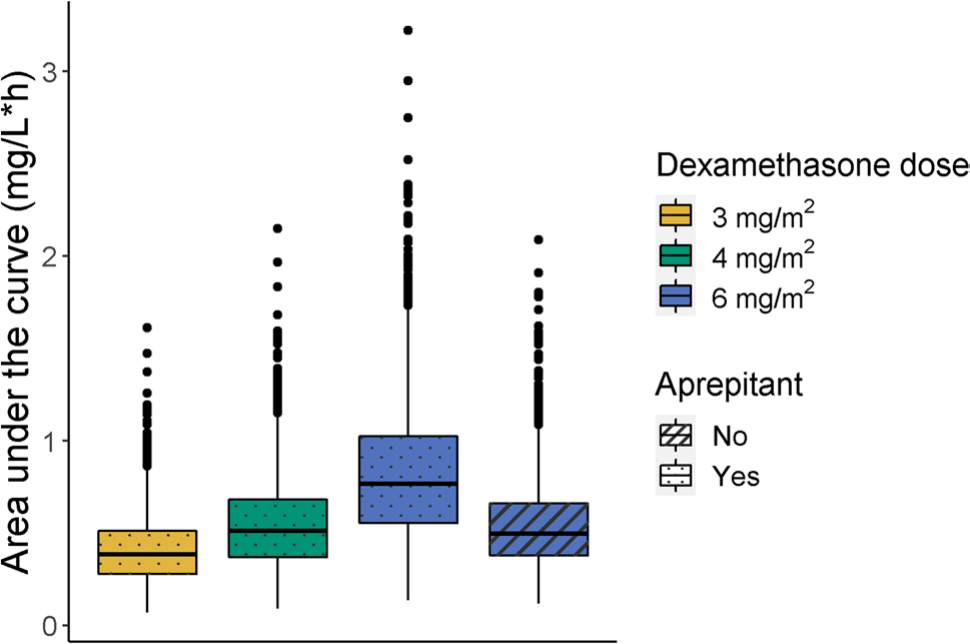
Dexamethasone area under the curve (AUC_t0-∞_) in simulated patients stratified for dose and aprepitant use. A single dexamethasone dose was simulated

Simulations were carried out and AUCs were calculated (Supplementary Table S2 and Fig. 2). Patients treated with 6 mg/m^2^ dexamethasone without aprepitant achieved a 54% higher median AUC_t0-∞_ than patients treated with 6 mg/m^2^ dexamethasone with aprepitant. When the patients with aprepitant were hypothetically treated with 3 mg/m^2^ dexamethasone (50% reduction), we observed that the dexamethasone AUC_t0-∞_ of patients with aprepitant was 23% lower than the AUC_t0-∞_ of patients without aprepitant. This again shows that a dose reduction of dexamethasone of 50% is not accurate to achieve comparable exposure. A dexamethasone dose of 4 mg/m^2^ (dose reduction of 33%) for patients receiving concurrent aprepitant was tested. This resulted in a comparable exposure between the two groups.

## Discussion

This population PK model is the first to describe the PK of dexamethasone (with and without aprepitant) as antiemetic agent in children. Dexamethasone PK was best described using a two compartment model, which is consistent with previously published models [22–25]. Firstly, a sole dexamethasone model was developed, including aprepitant as a categorical covariate. With concurrent aprepitant, dexamethasone CL changes with a proportional factor of 0.74 (95%CI 0.56–1.01), meaning a 26% lower CL with concurrent aprepitant. However, this solely model did not reflect clinical practice properly, since we expect the interaction to be dependent on the aprepitant plasma concentration. Therefore, we attributed the fact that the 95%CI of the interaction component includes 1 to a modelling artefact. An integrated dexamethasone-aprepitant model was developed subsequently, in order to accurately examine the influence of aprepitant on dexamethasone CL. We found that aprepitant reduced the dexamethasone CL by approximately 30%. This is not consistent with the results of studies in adults and current practice in children [11–14]. These studies all described a reduction of dexamethasone CL of approximately 50% or a doubling of the exposure to dexamethasone when combined with aprepitant.

At first, a possible explanation for the clinically relevant discrepancy with the results obtained in adults was thought to be related to age, since the rationale for dexamethasone dose reduction in children was extrapolated from adults. However, in our model development, no reason for testing age as covariate on any of the PK parameters was found. For this, we concluded that age has no obvious effect on the PK of dexamethasone or aprepitant.

Another possible explanation for the difference between adults and children could be found in the route of administration of dexamethasone. In several of the previously published articles, dexamethasone was administered orally [11, 13]. It was hypothesized that the presence of aprepitant reduces dexamethasone metabolism by inhibiting CYP3A4 enzymes in the gastro-intestinal (GI) tract, when dexamethasone is administered orally [15]. The inhibition of CYP3A4 enzymes in the GI-tract will lead to reduction of the first pass effect and thus bioavailability, which leads to a higher systemic dexamethasone exposure. When dexamethasone is administered intravenously, as in 95% of our pediatric patients, this effect will not play a role and only the systemic elimination of dexamethasone is influenced by concurrent use of aprepitant. However, two studies in adults did investigate the effect of aprepitant on intravenous dexamethasone, and also did find a reduction of the dexamethasone CL of approximately 50% [12, 14]. Since the drug-drug interaction with either oral or intravenous dexamethasone was never compared within a previous study, we initiated a future study to examine this. Within this study, patients will receive dexamethasone orally and intravenously in a cross-over design. We will include patients with and without aprepitant in a 1:1 ratio. The difference in PK and the bioavailability of dexamethasone and the influence of aprepitant will be studied in these cohorts (https://www.trialregister.nl/trial/8981).

In the current study, 11 children were excluded after informed consent was given, due to stopping dexamethasone because of off-target side effects like hypertension and/or behavioral problems. Although dexamethasone is strongly recommended in clinical antiemetic guidelines[1–3, 38], the side effects are a major issue in daily practice. Moreover, the appropriate dose to control CINV is intensively debated among different hospitals treating pediatric oncology patients. For example, in a survey among 36 children’s oncology institutions, two groups never administered dexamethasone. In the other 34 institutions, 29 different dexamethasone dosing schedules were used for children receiving HEC [39]. A recent systematic review aimed to describe all different dexamethasone doses studied for the prevention of chemotherapy-induced vomiting (CIV) in pediatric patients and their effects on achieving complete acute CIV control [40]. However, due to the heterogeneity of the studies and the wide variety in dosing schedules, no optimal dexamethasone dose to control acute CIV was found. Dosing regimens varied from 6 to 27 mg/m^2^/day in patients receiving HEC and 0.6–24 mg/m^2^/day in patients receiving MEC [40].

Translating the results of this study into clinical practice is challenging. On one hand, this current study shows that reduction of the intravenous dexamethasone dose with 50% in children might be too high, since we showed that aprepitant inhibits dexamethasone CL by approximately 30%. Based on the results of the current study, we advise to reduce the intravenous dexamethasone dose with 30% in children, when combined with aprepitant. However, as we already see many side effects of dexamethasone in our current HEC dose schedule, it is not very appealing to upgrade the current dexamethasone dose of 3 mg/m^2^ 4 times daily in presence of aprepitant. Triple therapy is the cornerstone of antiemetic treatment, however dexamethasone is regularly omitted due to side effects. Future studies should address dose reductions of dexamethasone, even as duration of antiemetic therapy in children, as many pediatric chemotherapy courses last much longer than the typical 3 day aprepitant regimen. For these future studies, we suggest to investigate a dose reduction of 30% in presence of aprepitant, and a dosing regimen within the dexamethasone dosing range (6–27 mg/m^2^/day) as described in the recent systematic review on the different dexamethasone dosing schedules worldwide [40].

Furthermore, our study underlines that extrapolating results from adults to children can be extremely difficult and should be done with caution. In our example, we found a clinically relevant discrepancy with the results in adults. That extrapolations from adults to children should be done with caution was previously described by Cella et al. [41]. They underlined that assuming a linear relationship between body weight and dose is not right for all drugs and that it can lead to either under- of overdosing. They suggested that dose recommendations for children should be derived from an integrated (model-based) analysis of PK data rather than from empiricism. This is in line with the results from our current study.

This study has several strengths. To start with, this is the first study that assessed the PK of dexamethasone as antiemetic agent and the influence of aprepitant on dexamethasone PK in children. Secondly, we used a rich sampling scheme and at least 5 blood samples were collected in 40 patients (62%). Furthermore, we developed an integrated dexamethasone and aprepitant model, where the inhibition of dexamethasone CL was dependent on the plasma concentration of aprepitant. This is closest to the real life situation in patients. To our knowledge, this was not tested before.

However, this study had some limitations too. Dexamethasone was administered intravenously in almost all patients, and therefore the difference of the effect of aprepitant on oral versus intravenous dexamethasone CL could not be identified. Because of this limitation, based on the current study, we can only give recommendations for intravenously administered dexamethasone in children.

Our second limitation refers to our planned sample size. We included 65 of the defined 90 patients. This was due to inclusion difficulties because of Covid-19 restrictions and the loss of eligible patients due to dexamethasone cancellation. The sample size of 90 patients was suggested in order to achieve a diverse population. However, we have shown in this analysis that we were able to accurately develop an integrated PK model with 65 patients. Moreover, we included our planned 5 children across all age groups except for the dexamethasone group of ≥ 6–12 years, in which only 2 children were sampled. The lower median age in the dexamethasone group compared to the dexamethasone plus aprepitant group was thought to be explained by the experience that older patients were treated with triple therapy more frequently than younger patients, due a higher occurrence of HEC chemotherapy courses.

Future studies are needed to optimize antiemetic control in pediatric oncology treatment. From this study we have learned the effect of aprepitant on dexamethasone CL in the pediatric oncology population. This important knowledge on the difference in CL compared to the adult population will be proceeded to the next phase in the optimization of antiemetic control. We are planning to conduct a randomized controlled trial in which we will study prolonged use of aprepitant to evaluate CINV control.

## Supplementary Information

Below is the link to the electronic supplementary material.

Supplementary file1 (DOCX 488 KB)

## Data Availability

Any reasonable requests to share data will be considered by the investigators. Data requests should be sent to the corresponding author.
