# Supplementary material for: Overestimation of the effect of (fos)aprepitant on intravenous dexamethasone pharmacokinetics requires adaptation of the guidelines for children with chemotherapy-induced nausea and vomiting
Source: Support Care Cancer. 2022 Oct 26;30(12):9991–9. doi: 10.1007/s00520-022-07423-6 (PMC9607815; doi:10.1007/s00520-022-07423-6)
Supplement: Supplementary file 1 — Supplementary file1 (DOCX 488 KB) [file 520_2022_7423_MOESM1_ESM.docx]

**Overestimation of the effect of (fos)aprepitant on intravenous dexamethasone pharmacokinetics requires adaptation of the guidelines for children with chemotherapy induced nausea and vomiting**

A. Laura Nijstad, Evelien de Vos-Kerkhof, Catherine F. Enters-Weijnen, Marianne D. van de Wetering, Wim J.E. Tissing, Matthijs M. Tibben, Hilde Rosing, Arief Lalmohamed, Alwin D.R. Huitema, C. Michel Zwaan

*Supportive Care in Cancer*

Corresponding author

Prof. dr. C. Michel Zwaan

Princess Máxima Center for Pediatric Oncology, Postbus 113, 3720 AC Bilthoven, The Netherlands

c.m.zwaan@prinsesmaximacentrum.nl

**Supplementary material**

**Supplementary Table S1** CYP3A4 substrates and/or –inhibitors. Patients using these CYP3A4 substrates and/or -inhibitors within seven days or CYP3A4 inducers within 30 days before the start of antiemetic therapy were excluded

| **CYP3A inhibitor (strong)** | **CYP3A inducer (strong)** |
| --- | --- |
| Boceprevir  Clarithromycin  Conivaptan  Grapefruit juice  Indinavir  Itraconazole  Ketoconazole  Lopinavir  Mibefradil  Nefazodone  Nelfinavir  Posaconazole  Ritonavir  Saquinavir  Telaprevir  Telithromycin  Voriconazole | Avasimibe  Carbamazepine  Phenytoin  Rifampin  St John’s wort |

**Supplementary Table S2** Sample scheme

| **Time after first dexamethasone/ (fos)aprepitant administration** | **t=0** | **t=0.5h** | **t=1-2h** | **t=4h** | **t=6h** | **t=12h** | **t=24h** |
| --- | --- | --- | --- | --- | --- | --- | --- |
| Sample | X | X | X | X | X | X | X |

*Sample scheme:*

Sample 1 (1 ml): t = 0 hour after first administration/end of infusion of dexamethasone and (fos)aprepitant

Sample 2 (1 ml): t = 0.5 hour after first administration/end of infusion of dexamethasone and (fos)aprepitant

Sample 3 (1 ml): t = 1-2 hours after first administration/end of infusion of dexamethasone and (fos)aprepitant

Sample 4 (1 ml): t = 4 hours after first administration/end of infusion of dexamethasone and (fos)aprepitant

Sample 5 (1 ml): t = 6 hours, just before the second administration of dexamethasone

Sample 6 (1 ml): t = 12 hours, just before the third administration of dexamethasone

Sample 7 (1 ml): t = 24 hours, just before the fifth administration of dexamethasone and second administration of (fos)aprepitant

**Supplementary Table S3** Dexamethasone area under the curve (AUC_t0-∞_) in mg/L*h in simulated patients stratified for dose and aprepitant use

|  | **With aprepitant** | | **Without aprepitant** | |
| --- | --- | --- | --- | --- |
|  | *Median* | *Range* | *Median* | *Range* |
| **Dexamethasone 6 mg/m^2^** | 0.768 | 0.135 – 3.311 | 0.498 | 0.117 – 2.090 |
| **Dexamethasone 4 mg/m^2^** | 0.512 | 0.090 – 2.207 | NA | |
| **Dexamethasone 3 mg/m^2^** | 0.384 | 0.068 – 1.656 | NA | |

*NA* not applicable

***
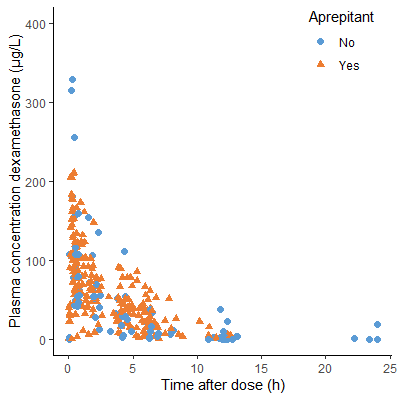
***

**Supplementary Figure S1** Dexamethasone plasma concentrations versus time after dose

***
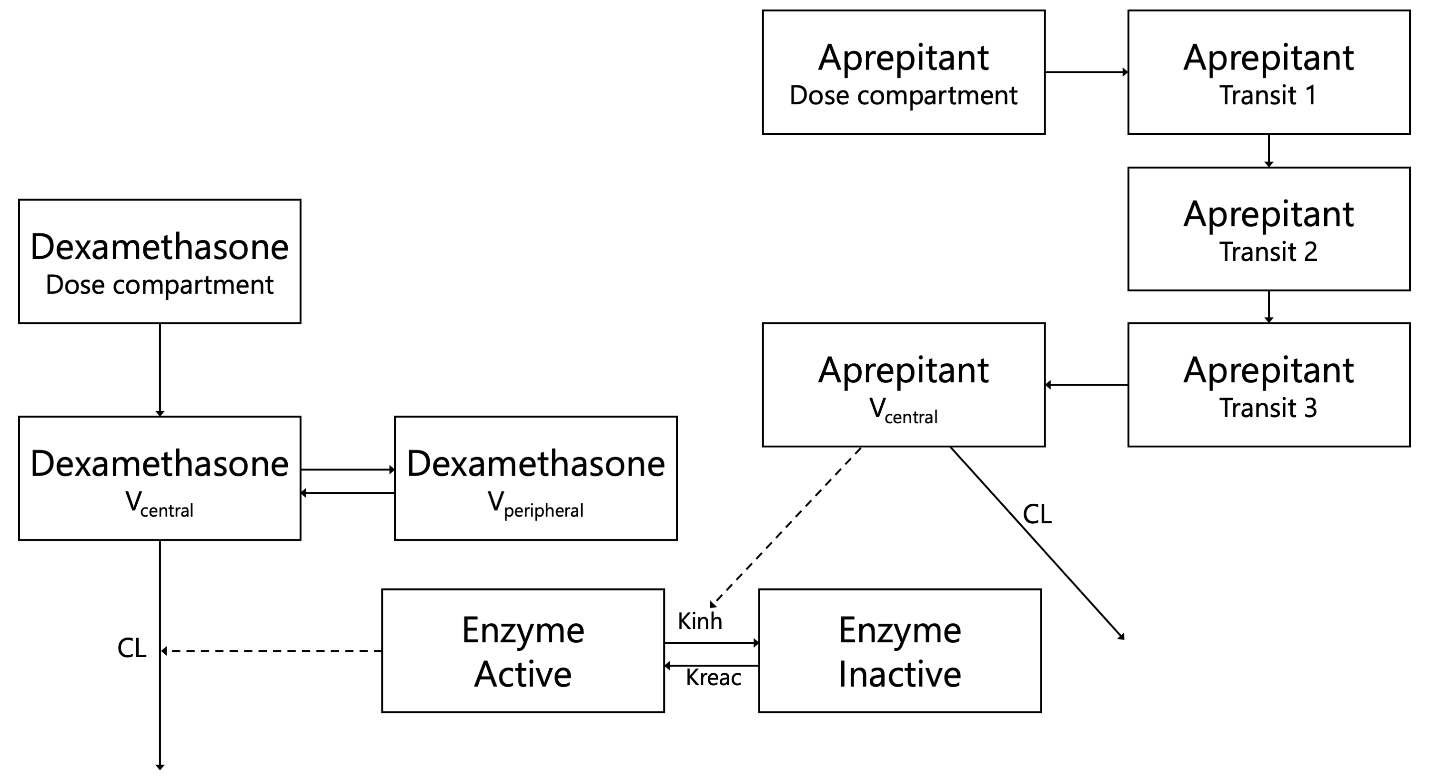
*Supplementary Figure S2** Graphical representation of the final integrated dexamethasone and aprepitant model

***
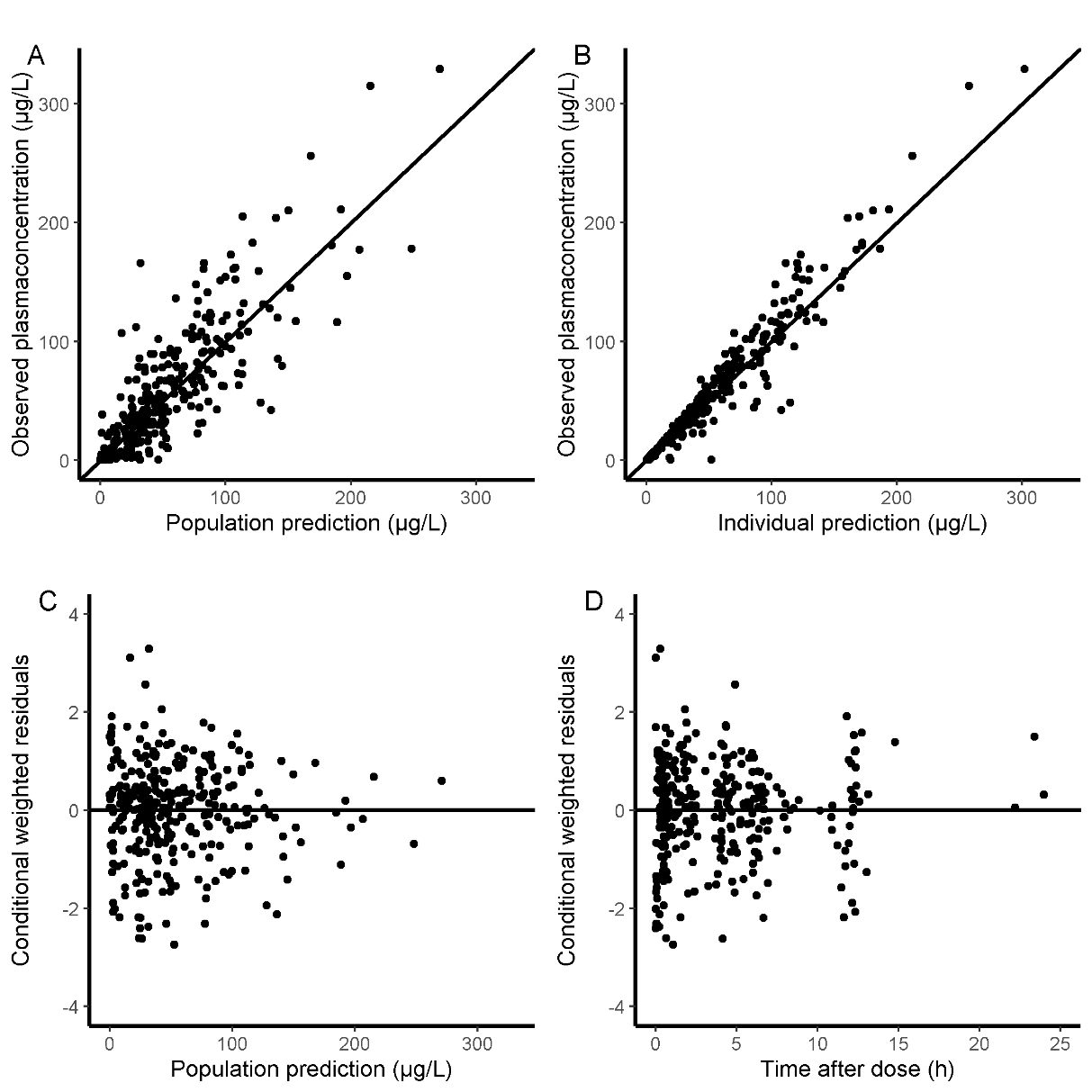
***

**Supplementary Figure S3** Goodness-of-fit plots dexamethasone

***
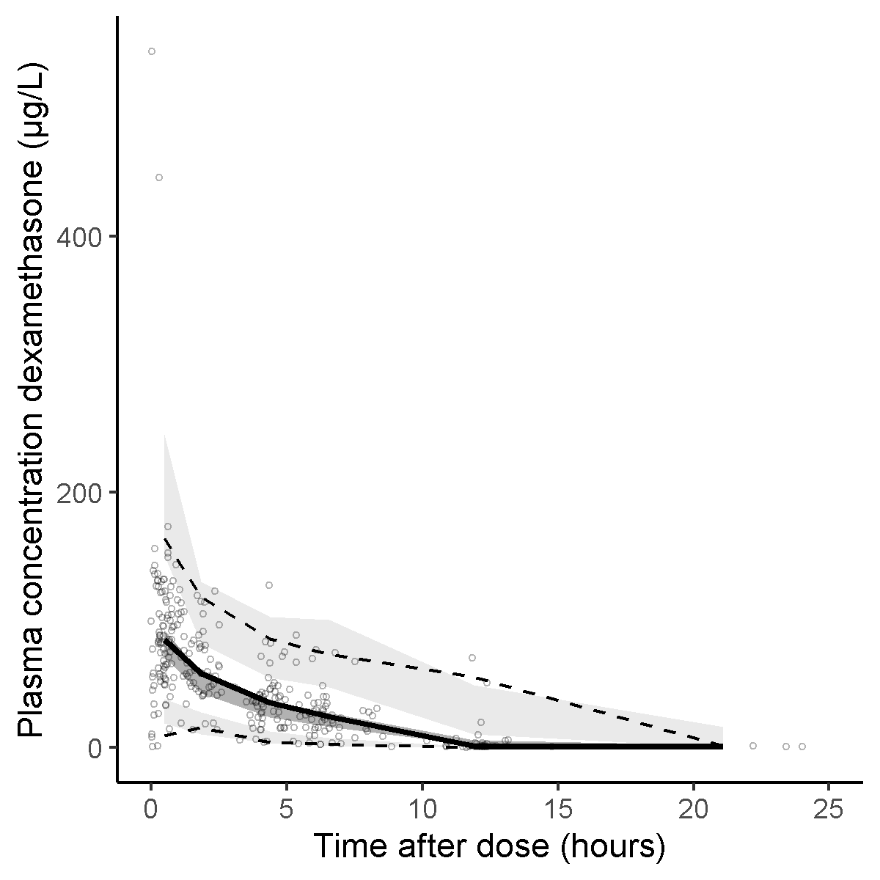
***

**Supplementary Figure S4** Prediction-corrected visual predictive check. Black lines depict the observed median (solid) and 2.5% and 97.5% percentile (dashed) concentrations. Dark- and light-grey areas represent 95% prediction intervals of the simulated mean and the 2.5 and 97.5% percentiles, respectively. Round dots represent observations.
